# Multi‐Targeting Carnosic Acid Kills Drug‐Resistant *Helicobacter pylori* With Narrow‐Spectrum Activity

**DOI:** 10.1002/advs.76080

**Published:** 2026-06-11

**Authors:** Yuefan Bai, Hongming Huang, Xudong Hang, Shanwei Hu, Qian Tong, Jingchen Xu, Jia Jia, Hongkai Bi

**Affiliations:** ^1^ NHC Key Laboratory of Tropical Disease Control School of Life Sciences and Medical Technology Hainan Medical University Haikou Hainan China; ^2^ Helicobacter Pylori Research Center Department of Pathogen Biology Jiangsu Key Laboratory of Pathogen Biology Nanjing Medical University Nanjing Jiangsu China; ^3^ Institute of Dermatology Hospital for Skin Diseases Chinese Academy of Medical Sciences & Peking Union Medical College Nanjing Jiangsu China; ^4^ Laboratory of Pathogen Biology State Key Laboratory of Cardiology and Research Center for Translational Medicine School of Medicine Shanghai East Hospital Tongji University Shanghai China; ^5^ Key Laboratory of Pathogen‐Host Interaction School of Medicine Tongji University Shanghai China

**Keywords:** carnosic acid, *Helicobacter pylori*, mode of action, narrow‐spectrum activity

## Abstract

*Helicobacter pylori* (*H. pylori*) is a significant global human pathogen intricately linked to gastritis, peptic ulcers, and gastric cancer. The escalating challenge of antimicrobial resistance and the adverse effects of conventional antibiotics on the gut microbiome necessitate the development of novel, targeted therapeutics. In this study, we demonstrate that carnosic acid (CA), a natural compound derived from traditional Chinese medicine, exhibits potent and specific anti‐*H. pylori* activity in vitro, with no detectable resistance observed after prolonged serial passaging. CA also displayed enhanced antibacterial efficacy under physiologically relevant acidic conditions, correlating with its strong inhibition of urease, a key colonization factor for *H. pylori*. Beyond urease suppression, CA acted through multiple mechanisms, including inhibiting biofilm formation and disrupting mature biofilms, impairing bacterial motility, and compromising cell membrane integrity. In vivo, the combination of CA and omeprazole achieved superior eradication in a mouse model of multidrug‐resistant *H. pylori* infection compared to standard triple therapy. Furthermore, CA treatment showed negligible toxicity to host tissues and minimal disruption to the diversity and composition of the gut microbiota. These findings position CA as a promising lead compound against drug‐resistant *H. pylori*, offering a multi‐targeting and microbiota‐friendly strategy to combat this pathogen.

## Graphical abstract

Carnosic acid selectively eradicates drug‐resistant *Helicobacter pylori* through a multi‐targeted mechanism involving urease inhibition, membrane disruption, and biofilm eradication. It exhibits no detectable resistance, potent in vivo efficacy, and minimal impact on gut microbiota, positioning it as a promising narrow‐spectrum lead compound against this pathogen.

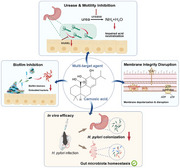

## 1 Introduction

*Helicobacter pylori* is a widespread human pathogen that infects over half of the global population [1]. *H. pylori* infection causes chronic gastritis and peptic ulcer disease, significantly increasing the risk of gastric mucosa‐associated lymphoid tissue lymphoma and gastric cancer [2, 3, 4]. Numerous studies have demonstrated that eradicating *H. pylori* can prevent relapse, accelerate peptic ulcer disease healing [5, 6], and, most critically, reduce gastric cancer incidence [7, 8, 9]. Triple or quadruple therapy, comprising two broad‐spectrum antibiotics (levofloxacin, metronidazole, clarithromycin, amoxicillin, and tetracycline) and a proton pump inhibitor (PPI), with or without the addition of bismuth, remains the recommended first‐line treatment for *H. pylori* infections [10]. However, eradication rates have declined due to the rapid emergence of antibiotic‐resistant *H. pylori* strains and poor patient compliance [11, 12]. In addition to genetic mutation, the ability of *H. pylori* to form biofilms contributes to drug resistance in conventional therapy [13, 14, 15]. Moreover, antibiotic use can nonspecifically eliminate commensal bacteria and disturb the microbiota balance [16, 17]. These alterations in the gut microbiota may disrupt host metabolism and immunity, increasing disease risk [18]. Therefore, developing innovative and efficient anti‐*H. pylori* agents with low resistance potential, antibiofilm activity, and minimal impact on intestinal flora is critical for improving treatment success rates in clinical practice.

Targeting virulence factors presents an alternative strategy for treating infections caused by antibiotic‐resistant bacteria. This approach may impose less evolutionary pressure on the development of resistance, as most virulence factors are not essential for bacterial growth. Several virulence factors play crucial roles in *H. pylori* colonization and virulence, including flagella [19], urease [20, 21], adhesins, and virulence proteins such as CagA [22, 23] and VacA [24]. Among them, compounds with both anti‐motility and anti‐urease activities have drawn considerable attention. The motility of *H. pylori* is mediated by a unipolar bundle of spiral‐shaped flagella. The flagella enable the bacteria to move through the viscous mucus lining of the stomach, reach gastric epithelial cells, and initiate an infection [25]. Urease catalyzes the hydrolysis of urea into ammonia and carbon dioxide, which helps the bacteria survive during short‐term exposure to the extremely low pH levels in the gastric lumen [26]. These two factors are essential for the colonization and establishment of chronic *H. pylori* infections [27, 28]. Effective anti‐motility and urease inhibitors hold great promise for reducing the bacterial load, minimizing mucosal irritation, and thus are of significant research interest.

Traditional Chinese medicines (TCM) have long served as a rich source of lead compounds. Many TCM monomers exhibit potent antimicrobial activity, either alone or in synergy with antibiotics. For example, quercetin inhibits *Staphylococcus aureus* growth [29, 30], and piperine from *Piper nigrum* shows synergistic effects with rifampin against *Mycobacterium tuberculosis* [31]. Natural compounds such as berberine and allicin have demonstrated antibacterial activity against *H. pylori* and can attenuate inflammation in gastric disease [32, 33, 34, 35]. However, research on their specific targets remains preliminary, highlighting an area of considerable potential.

*Salvia officinalis L*. (sage) and *Rosmarinus officinalis L*. (rosemary) are members of the *Lamiaceae* family within the *Lamiales* order [36]. As well‐known TCMs, they are highly valued for their therapeutic properties and pharmacological effects. Extracts of sage and rosemary have been reported to possess anti‐inflammatory and memory‐enhancing properties [37]. They can also stimulate blood circulation, thereby alleviating adrenal and segmental rheumatic muscle discomfort [38]. Carnosic acid (CA), a phenolic diterpene compound, is one of the primary bioactive constituents of rosemary and sage. It was first identified by Linde in *Salvia officinalis L* [39]. Over the past decade, research has demonstrated that CA possesses multiple bioactive properties, including antioxidant [40], anti‐inflammatory [41, 42], and anticancer activities [43]. Notably, CA derivatives also exhibit gastroprotective properties [44, 45], prompting our investigation into its potential activity against *H. pylori*. A recent study using molecular docking predicted that CA might inhibit *H. pylori* glucose‐6‐phosphate dehydrogenase (G6PD), based on its presence in *Salvia officinalis* extracts with reported anti‐*H. pylori* activity [46]. However, the direct antibacterial effects of purified CA, its bactericidal mechanism, and its in vivo efficacy against *H. pylori* have not been systematically investigated.

In this study, we report that carnosic acid demonstrates potent bactericidal activity against both drug‐sensitive and multidrug‐resistant *H. pylori* strains in vitro and in vivo. Beyond its direct antimicrobial effect, CA significantly inhibits key virulence traits: urease activity, biofilm formation, and bacterial motility. Furthermore, CA exhibits a low propensity for resistance development and causes minimal disturbance to the gut microbiota—distinct advantages over conventional antibiotics. These multi‑mechanistic and microbiota‑sparing properties establish CA as a promising lead compound for the eradication of multidrug‐resistant *H. pylori*.

## 2 Results

### 2.1 CA Demonstrates Selective Anti‐*H. pylori* Efficacy In Vitro

**FIGURE 1:**
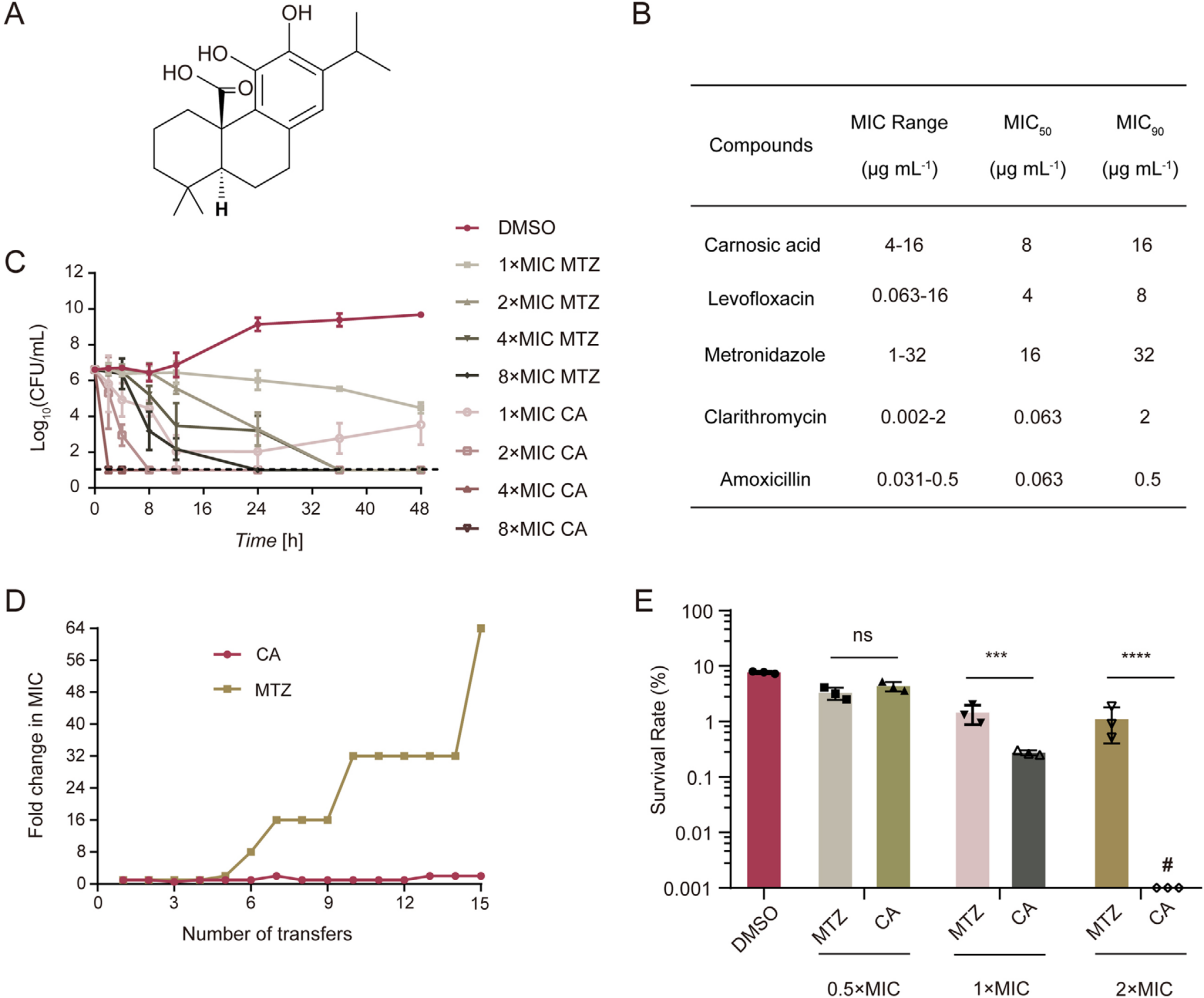
In vitro anti‐*H. pylori* activity of carnosic Acid (CA). (A) Chemical structure of CA. (B) Antibacterial activities of CA and four clinical antibiotics against 35 *H. pylori* standard strains and drug‐resistant clinical isolates. (C) Bactericidal kinetics of CA against *H. pylori* G27. The MICs of CA and metronidazole (MTZ) were 16 and 2 µg/mL, respectively. At 4× and 8× MIC, CA reduced viable counts below the detection limit (dotted line) within 2 h, resulting in indistinguishable killing curves. (D) Development of resistance to CA and MTZ in *H. pylori* G27. Fold change is determined as the normalized ratio of the MIC obtained for a certain subculture to the MIC recorded at first exposure. (E) The survival rate of *H. pylori* G27 after incubation with CA or MTZ for 30 min at pH 2.5. The survival rate is the ratio of the number of viable bacteria in acidic conditions (pH 2.5) to the number of viable bacteria in neutral conditions (pH 7.0). Data are presented as mean ± s.d. from three independent experiments. Statistical significance was determined using one‐way ANOVA followed by Tukey's multiple comparisons test. #, not detected; ns, not significant (*p* > 0.05); ***, *P*<0.001; ****, *P*<0.0001.

The anti‐*H. pylori* potential of CA (Figure 1A) was initially evaluated using a broth micro‐dilution assay against the standard strain G27. The minimal inhibitory concentration (MIC) value of CA against this strain was determined to be 16 µg mL^−^^1^. Subsequently, a comprehensive evaluation against a panel of 41 *H. pylori* strains (including 38 single antibiotic‐resistant and multidrug‐resistant clinical isolates) confirmed its potent activity, with MIC_50/90_ values of 8/16 µg mL^−1^ (range: 4–16 µg mL^−1^) (Figure 1B; Table S1), indicating a lack of cross‐resistance. To assess its spectrum of activity, we measured the MIC values of CA against a total of 29 bacterial and 4 fungal species. CA exhibited relatively low antimicrobial activity against only a few other strains, such as *Staphylococcus aureus* and *Moraxella catarrhalis* (MIC = 16 µg mL^−^^1^) (Table 1), indicating that it is a narrow‐spectrum antimicrobial with selective efficacy against *H. pylori*.

**TABLE 1:** Antimicrobial spectrum of CA.

	Strains	MIC [µg mL^−1^]			
		CA^a^	AMB^b^	AMP^c^	VAN^d^
Gram Positive Bacteria	*Staphylococcus aureus* ATCC25923	16	—^e^	0.125	1
	*Staphylococcus hemolytic* ATCC29970	16	—	8	2
	*Bacillus subtilis* 168	32	—	—	0.25
	*Bacillus cereus* ATCC14579	32	—	16	2
	*Listeria monocytogenes* EGD‐e	32	—	0.125	2
	*Streptococcus pneumoniae* ATCC49619	128	—	2	2
	*Enterococcus faecalis* ATCC29212	32	—	1	2
	*Enterococcus faecium* ATCC19434	64	—	1	1
	*Bifidobacterium longum* ATCC15697	128	—	—	0.5
	*Propionibacterium acnes* ATCC11827	>128	—	>128	16
	*Lactobacillus plantarum* ATCC8014	32	—	<1	32
Gram Negative Bacteria	*Actinobacillus actinomycetes* D7S‐1	64	—	2	2
	*Campylobacter jejuni* NCTC11168	>128	—	4	2
	*Haemophilus haemolyticus* ATCC49766	32	—	16	1
	*Escherichia coli* ATCC25922	>128	—	4	—
	*Pseudomonas aeruginosa* PAO1	>128	—	>128	—
	*Klebsuella pneumoniae* ATCC35657	>128	—	>128	—
	*Acinetobacter baumannii* ATCC19606	>128	—	128	—
	*Salmonella typhimurium* ATCC14028	>128	—	1	—
	*Enterobacter cloacae* ATCC13047	>128	—	>128	—
	*Stenotrophomonas maltophilia* ATCC51331	128	—	>128	—
	*Shigella dysenteriae* Sd197	>128	—	>128	—
	*Prevotella intermedia* ATCC25611	32	—	—	4
	*Fusobacterium nucleatum* ATCC25586	128	—	<1	2
	*Moraxella catarrhalis* ATCC25238	16	—	2	16
	*Proteus mirabilis* ATCC29906	128	—	>128	—
	*Bacteroides fragilis* ATCC25285	32	—	2	2
	*Akkermansia muciniphila* ATCC‐BAA‐835	128	—	<1	16
Mycobacterium	*Mycobacterium smegmatis* ATCCC607	>128	—	—	—
Fungus	*Candida albicans* SC5314	64	1	—	—
	*Aspergillus fumigatus* Af293	>128	<0.25	—	—
	*Malassezia syrmpodiais* CBS7222	64	1	—	—
	*Malassezia globosa* CBS7966	64	1	—	—

^a^ CA, Carnosic acid.

^b^ AMB, Amphotericin B.

^c^ AMP, Ampicillin.

^d^ VAN, Vancomycin.

^e^ —, Not determined.

We next investigated the bactericidal kinetics of CA against *H. pylori* strain G27. CA exhibited rapid bactericidal activity, achieving a 99.9% reduction in colony‐forming units (CFUs) within 4 h at 2×MIC (32 µg mL^−1^). At higher concentrations (4× and 8×MIC), the bacterial counts were reduced to below the detection limit within 2 h. Complete killing, with no recoverable colonies after 48 h, was observed at all concentrations except 1×MIC (Figure 1C). These results suggest that the bactericidal action of CA is concentration‐dependent. In contrast, metronidazole (MTZ) killed strain G27 at a much slower rate. A critical advantage of CA is its low propensity for resistance development. During continuous serial passaging for 60 days, the MIC of CA against strain G27 remained unchanged. Conversely, the MIC of MTZ increased 64‐fold after only 15 passages (Figure 1D), underscoring CA's stability against resistance.

Given that *H. pylori* colonizes the acidic gastric environment, we investigated CA's efficacy under acidic conditions (pH 2.5, 10 mM urea). When tested at equivalent MIC‐fold concentrations, CA elicited significantly more potent bactericidal effect than MTZ. Notably, at 2×MIC, the bacterial count in the CA‐treated group was reduced to the detection limit, three orders of magnitude lower than that in the MTZ‐treated group (Figure 1E). These findings demonstrate that CA retains superior anti‐*H. pylori* activity under physiologically relevant acidic conditions.

### 2.2 CA Exhibits Potent *H. pylori* Urease Inhibitory Activity

**FIGURE 2:**
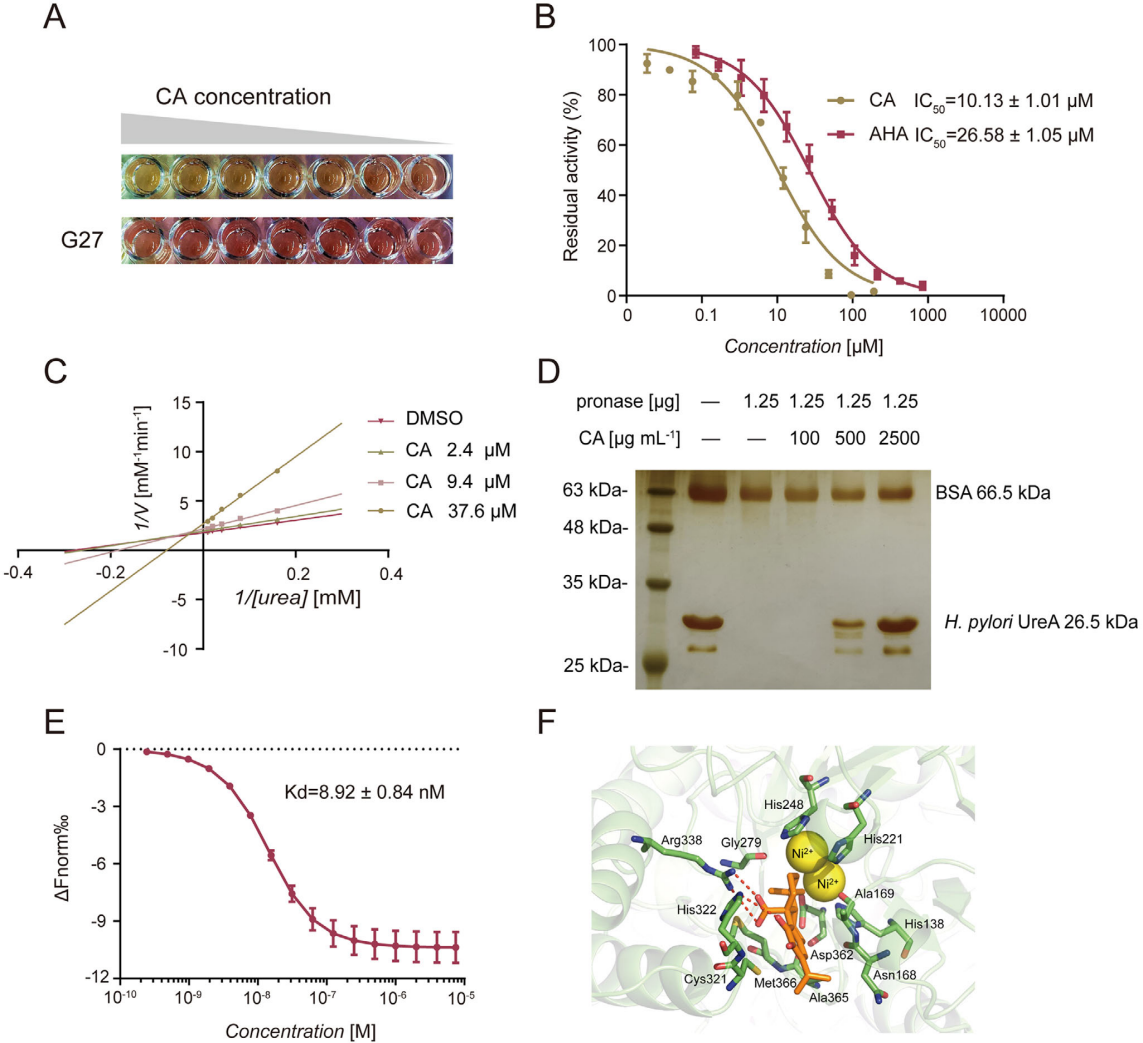
Inhibition of Urease activity by CA. (A) The inhibitory activity of the compounds against *H. pylori* urease was determined using Christensen urea medium. As the concentration of the compound increases, the inhibitory effect is progressively increased, accompanied by a corresponding lightening of the culture medium's color. (B) IC_50_ determination of *H. pylori* urease inhibition by CA using the Berthelot method. AHA, acetohydroxamic acid. All data are presented as mean ± s.d. from three independent experiments. (C) Double‐reciprocal plots of 1/V versus 1/[urea] at different inhibitor concentrations demonstrate that CA exhibits a mixed‐type inhibition against *H. pylori* urease, with *K*_i_ values of 7.63 ± 1.36 µM. (D) Silver staining was performed to evaluate the binding affinity between CA and HpUreA (26.5 kDa), with BSA (66.5 kDa) serving as a control protein that was resistant to degradation by pronase. (E) MST measurements of CA binding to UreA. Normalized fluorescence (Δ*F*norm‰) from three independent experiments, with s.d. shown, was plotted against the concentration of CA. Data were analyzed with the *K*_d_ model using the MO. Affinity. (F) CA (orange sticks) is docked into *H. pylori* (green sticks) urease using Schrödinger 2015 software. Red dotted lines indicate hydrogen bonds.

*H. pylori* relies on urease to neutralize gastric acid, a critical step for colonization and pathogenesis. We then investigated whether CA could inhibit this key enzyme to modulate its killing efficacy under acidic conditions. Using a qualitative Christensen's urea broth assay [47], we observed a dose‐dependent inhibition of urease by CA, indicated by the color change of the medium from red (alkaline/active urease) to yellow (neutral/inhibited urease) (Figure 2A). Quantitative analysis using the Berthelot method [48] confirmed CA as a highly potent urease inhibitor, with an IC_50_ of 10.13 ± 1.01 µM. This inhibitory activity was superior to the standard inhibitor acetohydroxamic acid (AHA; IC_50_ = 26.58 ± 1.05 µM) (Figure 2B). Among a panel of related rosemary compounds (Figure S1), only carnosol exhibited comparable activity, while all others were significantly weaker (Figure S2).

Next, we investigated the inhibition mechanism of CA by plotting the data using the Lineweaver‐Burk method. The plots clearly intersect in the second quadrant (Figure 2C), indicating mixed inhibition toward *H. pylori* urease. This suggests that CA can bind to both the active site of the enzyme and the enzyme‐substrate complex, thereby inhibiting the enzyme's activity. The *K*_i_ value, calculated directly from Dixon plots, was 7.63 ± 1.36 µM, demonstrating the high affinity of CA for *H. pylori* urease.

Both drug affinity responsive targeting stability (DARTS) and microscale thermophoresis (MST) assays were employed to further validate the direct interaction between CA and the UreA subunit of *H. pylori* urease. In the DARTS assay, small molecules can protect target proteins from degradation by pronase [49]. As shown in Figure 2D, CA significantly attenuated the proteolysis of *H. pylori* UreA induced by pronase. Bovine serum albumin (BSA), used as a loading control, was not degraded by pronase and remained unaffected by CA treatment. Consistent with this, the equilibrium dissociation constant (*K*_d_) value for CA and *H. pylori* UreA was determined to be 8.92 ± 0.84 nM from MST assay (Figure 2E), suggesting a relatively strong binding affinity.

To further elucidate the binding mode of CA with *H. pylori* urease, we performed molecular docking. The model positions CA within the enzyme's active site, where it coordinates the two essential Ni^2^^+^ ions. A key hydrogen bond is formed between the carboxyl group of CA and Arg338 of UreA (Figure 2F). Notably, the Ni^2^^+^ ions interact with the doubly substituted methyl groups on the ring of CA rather than with polar groups. This unique binding pose within the active site provides a structural rationale for the observed mixed‐type inhibition.

### 2.3 CA Exhibits Anti‐Biofilm and Anti‐Motility Activity

**FIGURE 3:**
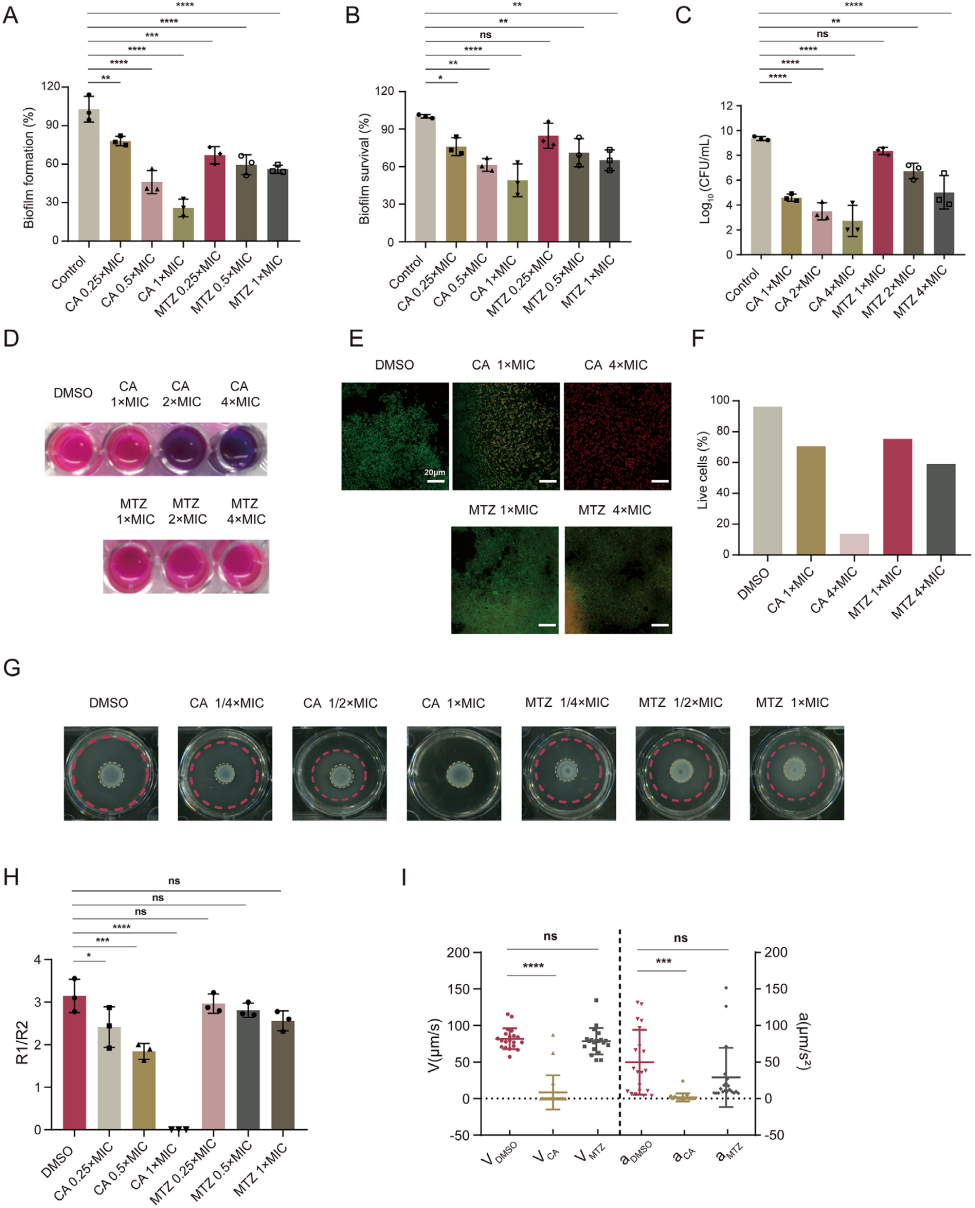
Anti‐biofilm and anti‐motility activity of CA. (A‐F) CA exhibits anti‐biofilm activity. The ability of CA to inhibit biofilm formation (A) and to disrupt preformed biofilms (B) of *H. pylori* G27 was determined using crystal violet staining. The effect of CA on the cell viability of preformed biofilms was evaluated using viable colony counting (C), Alamar Blue staining method (D), and SYTO9/PI double staining (E). Scale bar, 20 µm. The percentage of live cells (green/total cells) was calculated using Image J (F) based on the SYTO9/PI staining images shown in panel E. (G‐I) CA exhibits anti‐motility activity. The inhibitory effect of CA on *H. pylori* motility was visually demonstrated using soft agar medium containing 0.35% agar (G). The motility of *H. pylori* after treatment with different compounds was quantified by calculating the ratio of the halo radius (R1, Red dotted circle) to the bacterial growth zone radius (R2, Yellow dotted circle) (H). Bacterial speed and acceleration within the field of view were analyzed using CSRT algorithm implemented in Python (I). DMSO and MTZ served as negative and positive controls, respectively. Data are presented as mean ± s.d. from three independent experiments. Statistical significance was determined using one‐way ANOVA followed by Dunnett's multiple comparisons test. ns, not significant (*p* > 0.05); *, *p* < 0.05; **, *p* < 0.01; ***, *p* < 0.001; ****, *p* < 0.0001.

Targeting biofilm formation represents a promising strategy to enhance *H. pylori* eradication [50, 51]. We evaluated the anti‐biofilm potential of CA using crystal violet assays. CA demonstrated a superior capacity to inhibit biofilm formation compared to metronidazole (MTZ), achieving 75% and 45% inhibition of *H. pylori* G27 biofilms at their respective 1×MIC concentrations (Figure 3A). Furthermore, CA was significantly more effective than MTZ at disrupting pre‐established mature biofilms (Figure 3B).

The potent antibiofilm activity of CA was further confirmed through viable cell counts. Treatment of biofilms with CA (16–64 µg mL^−^^1^) resulted in a substantial reduction of 4.3 to 6.6 Log10 (CFU/mL) compared to the DMSO control (Figure 3C). While MTZ showed moderate activity, it was less effective than CA. Metabolic activity, assessed by Alamar blue staining, confirmed that CA at 2×MIC effectively eradicated bacteria within biofilms (indigo blue), whereas MTZ at 4×MIC failed to eliminate the bacteria (pink) (Figure 3D). This finding was further supported by confocal microscopy following SYTO 9/PI staining. While the control biofilms exhibited predominantly viable (green) cells, treatment with CA at 4×MIC (64 µg mL^−^^1^) resulted in near‐complete bacterial cell death, as indicated by pervasive red PI fluorescence (Figure 3E). Quantitative image analysis further confirmed this observation, showing a marked increase in the proportion of dead cells and a corresponding reduction in viable cells upon CA treatment (Figure 3F). Collectively, these results indicate that CA not only inhibits biofilm formation but also effectively kills the embedded *H. pylori* cells.

We next investigated the effect of CA on *H. pylori* motility, a critical virulence factor for colonization. Soft‐agar assays revealed a concentration‐dependent inhibition of bacterial swimming by CA (Figure 3G; growth area indicated by yellow circles, swimming halos indicated by red circles). The motility was completely abolished at the 1×MIC concentration of CA, in contrast to the minimal effect observed with MTZ (Figure 3H). To quantify this effect on individual bacteria, we analyzed cell movement using dark‐field microscopy and tracking algorithms. At a sub‐inhibitory concentration (1/2×MIC), treatment with CA significantly reduced bacterial speed and acceleration compared to both DMSO and MTZ treatments (Figure 3I). These findings demonstrate that CA effectively impairs a key mechanism of *H. pylori* pathogenesis.

### 2.4 CA Exerts Bactericidal Activity by Disrupting Bacterial Membrane Integrity and Function

**FIGURE 4:**
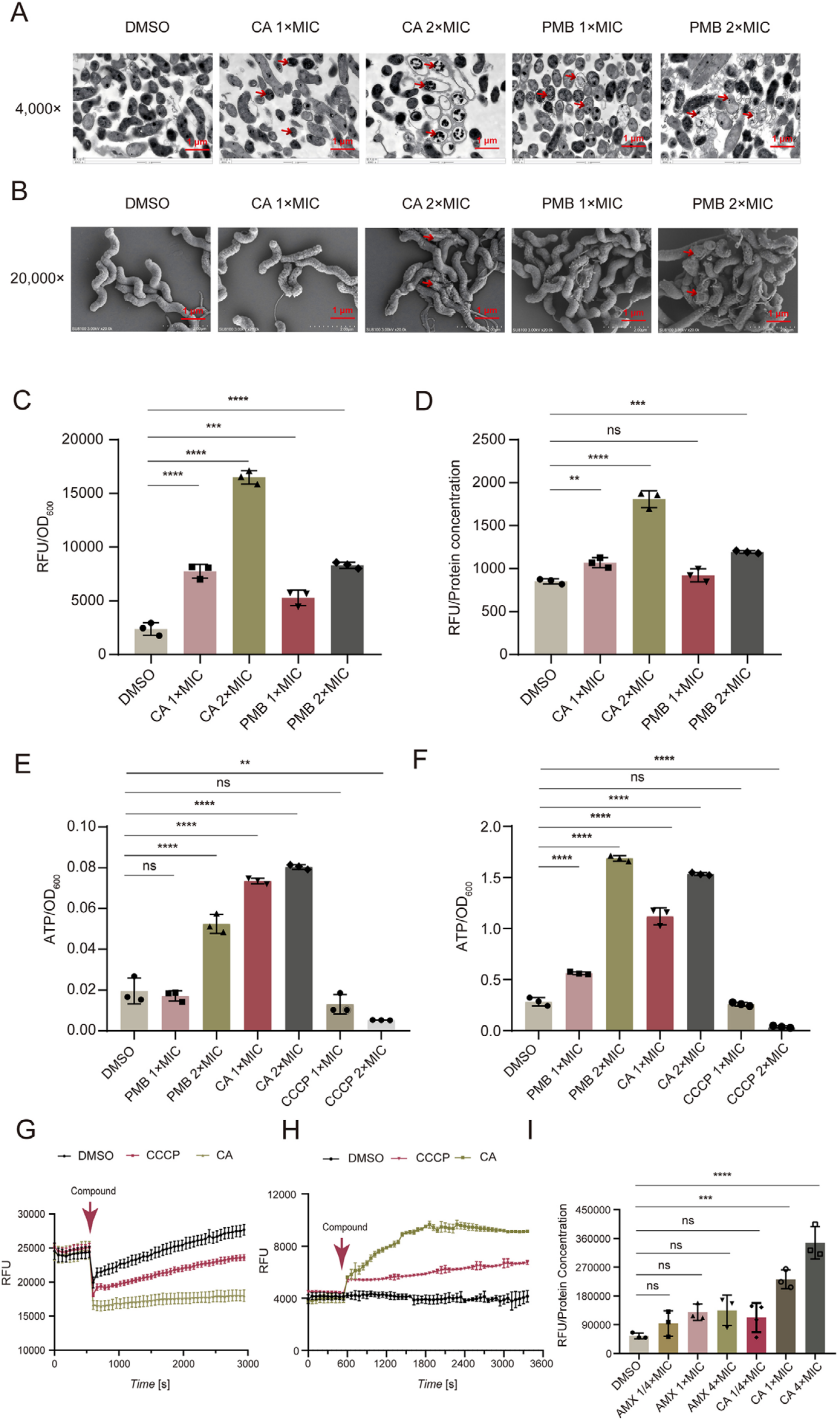
Effect of CA on the morphology and function of *H. pylori* cell membranes. (A, B) Morphological alterations in *H. pylori* G27 cells following exposure to CA were observed by TEM (A) and SEM (B). DMSO and PMB served as negative and positive controls, respectively. (C) Decreased outer membrane integrity following CA treatment was determined by NPN staining. (D) Increased membrane permeability following CA treatment was determined by PI staining. (E, F) Extracellular ATP content (E) and intracellular ATP content (F) were measured using the microbial cell viability assay kit. PMB was used as a positive control for disrupting cell membranes and causing intracellular ATP leakage, while carbonyl cyanide m‐chlorophenyl hydrazone (CCCP) served as a positive control for inhibiting ATP synthesis. (G, H) The effects of CA treatment on the primary components of the PMF, ΔpH (G) and Δψ (H). Fluorescent probes BCECF‐AM and DiSC_2_(3) were used for detection, respectively. CA at 32 µg mL^−1^ was used in both assays, while CCCP was applied as a positive control at 32 µg mL^−1^ for ΔpH measurement and 0.25 µg mL^−1^ for Δψ measurement. (I) The effect of CA on ROS accumulation in *H. pylori* after 4 h of treatment, whereas amoxicillin (AMX) treatment does not induce ROS accumulation. Data are presented as mean ± s.d. from three independent experiments. Statistical significance was determined using one‐way ANOVA followed by Dunnett's multiple comparisons test. ns, not significant (*p* > 0.05); **, *p* < 0.01; ***, *p* < 0.001; ****, *p* < 0.0001.

To elucidate the bactericidal mechanism of CA, we first investigated its effect on the cellular morphology of *H. pylori* using transmission electron microscopy (TEM) and scanning electron microscopy (SEM). Following a 1‐h exposure, DMSO‐treated bacteria exhibited intact cell walls and densely packed cytoplasm, maintaining their typical spiral or rod‐like morphology. In contrast, treatment with CA resulted in pronounced ultrastructural alterations, characterized by relatively severe cellular damage, including cytoplasmic vacuolization, partial separation of the outer and plasma membranes, and evidence of intracellular content leakage (Figure 4A). Consistent with the TEM observations, SEM analysis revealed that *H. pylori* cells treated with CA led to clear membrane disruption and structural collapse, displaying morphological features similar to those observed in the polymyxin B (PMB)–treated positive control (Figure 4B). These structural changes were accompanied by functional impairment of the bacterial membrane. To specifically assess whether CA disrupts the outer membrane (OM) barrier of *H. pylori*, an N‐phenyl‐1‐naphthylamine (NPN) uptake assay was performed. As NPN fluorescence increases upon entry into hydrophobic environments following OM damage, this assay provides a direct measure of OM permeability. Treatment with CA resulted in a significant, dose‐dependent increase in NPN fluorescence compared with the DMSO control (Figure 4C), indicating that CA effectively compromises the integrity of *H. pylori* OM barrier.

Consistent with OM permeabilization, overall membrane permeability was further increased in CA‐treated cells, as determined by propidium iodide (PI) staining (Figure 4D). We further probed membrane integrity by measuring the release of intracellular ATP. Treatment of H. pylori G27 with CA resulted in a dose‐dependent increase in ATP release into the medium, comparable to the effect of the membrane‐disrupting agent PMB (Figure 4E). This demonstrates a direct impairment of the plasma membrane's barrier function. Interestingly, exposure to CA elicited an increase in cellular ATP levels (Figure 4F), a phenomenon previously associated with antibiotic‐induced metabolic perturbation [52]. In contrast, treatment with the oxidative phosphorylation uncoupler carbonyl cyanide m‐chlorophenylhydrazone (CCCP) significantly suppressed ATP synthesis, serving as a positive control for respiratory inhibition.

To determine whether CA‐induced membrane disruption is specific to H. pylori, comparative experiments were performed using two CA‐insensitive Gram‐negative bacterial strains, Escherichia coli ATCC 25922 and Pseudomonas aeruginosa PAO1. PI uptake assays conducted within CA exposure showed no significant increase in fluorescence in either strain (Figure S3A). Consistently, TEM analysis revealed no apparent alterations in membrane architecture following CA treatment (Figure S3B). These findings indicate that CA does not broadly disrupt Gram‐negative bacterial membranes, but instead exhibits selective membrane activity toward H. pylori.

Given the evidence of membrane damage, we investigated the impact of CA on the proton motive force (PMF), a key driver of bacterial energetics. Using the fluorescent probe BCECF‐AM, we observed that CA dissipated the transmembrane pH gradient (ΔpH), similar to the positive control CCCP (Figure 4G). The PMF is maintained by a compensatory relationship between ΔpH and the membrane potential (Δψ) [53]. Measurement of Δψ using the DiSC_2_(3) dye revealed that CA treatment also led to a collapse of the membrane potential, accompanied by increased fluorescence (Figure 4H).

The disruption of membrane homeostasis and energy metabolism is recognized as a trigger for the accumulation of reactive oxygen species (ROS) [54]. Consistent with the action of other bactericidal antibiotics, CA treatment elevated intracellular ROS levels in a dose‐dependent manner (Figure 4I). Fluorescence microscopy further confirmed increased ROS levels in CA‐treated cells compared to the DMSO control (Figure S4), suggesting that oxidative stress acts in a feed‐forward manner to exacerbate the initial membrane damage. In summary, CA kills *H. pylori* by directly targeting and disrupting the bacterial membrane, leading to dissipation of the PMF, and ROS‐induced amplification of cellular damage.

### 2.5 CA Kills *H. pylori* In Vivo

**FIGURE 5:**
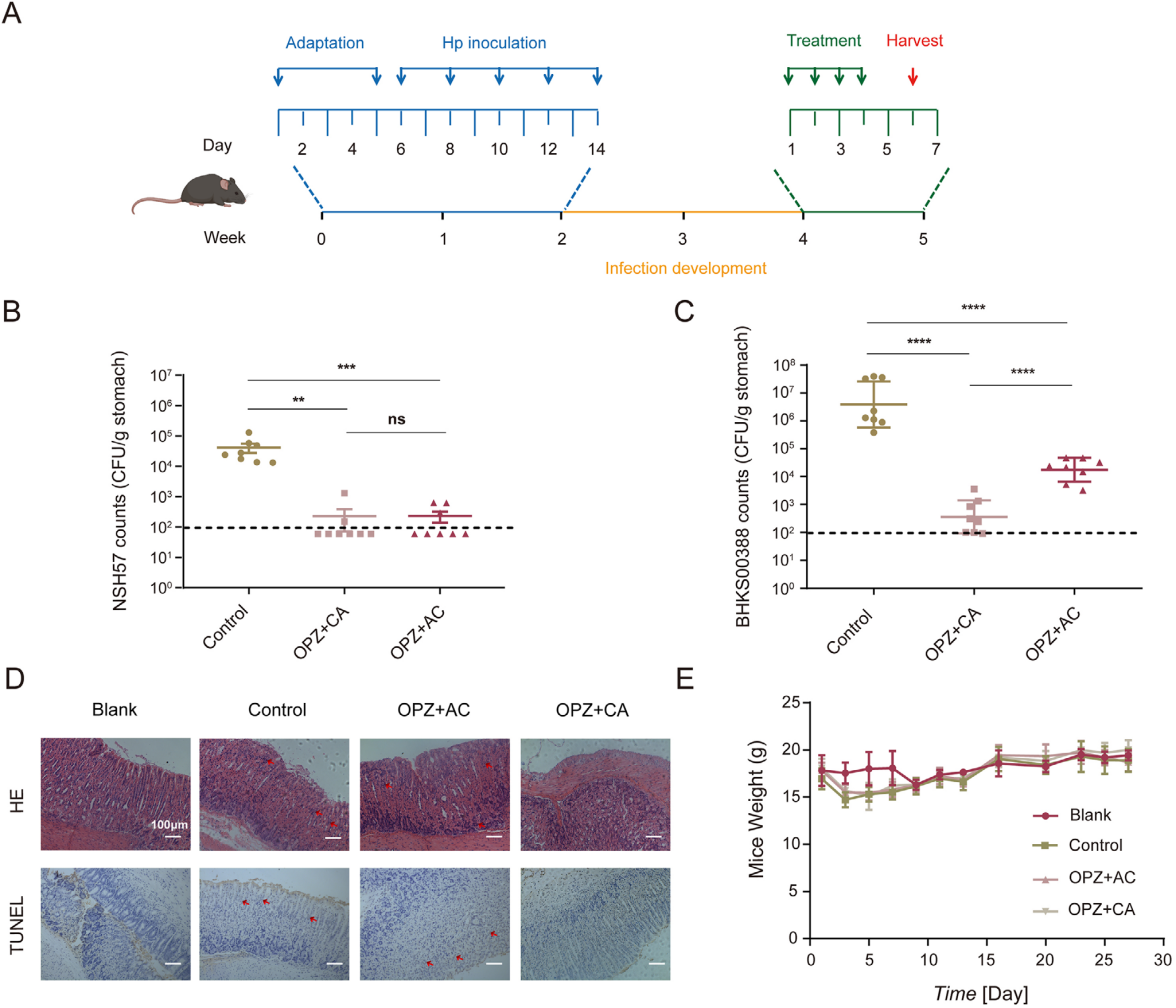
In vivo efficacy of CA against *H. pylori* infection in mouse models. (A) A schematic diagram of *H. pylori* inoculation, infection development, and treatments in C57BL/6J mice. (B, C) Quantification of bacterial burdens in the stomachs of mice infected with the *H. pylori* standard strain NSH57 (B) or strain BHKS00388 (C), euthanatized 48 h after the last treatment with CMC‐Na (control), OPZ + CA, and triple therapy (OPZ + AC). Data are represented as mean ± s.d. (n = 8 mice/group). If no colonies were detected, the bacterial burden was calculated using the limit of detection (10^2^ CFU/g stomach). Statistical differences among groups in panels B and C were analyzed using one‐way ANOVA followed by Tukey's multiple comparisons test. ns, no significant difference (*p* > 0.05); **, *p* < 0.01; ***, *p* < 0.001; ****, *p* < 0.0001. (D) Micrographs of stomach biopsies stained with H&E (hematoxylin and eosin) and TUNEL (terminal deoxynucleotidyl transferase dUTP nick end labeling) after the indicated treatments. Scale bar, 100 µm. (E) The trend in body weight changes in each group of mice during the infection and treatment process.

Given its potent in vitro anti‐*H. pylori* activity, we used *H. pylori*‐infected mouse models to evaluate the in vivo therapeutic potential of CA. Mice were infected with *H. pylori* NSH57 [55], a mouse‐adapted derivative of the G27 strain, via oral gavage every other day for a total of five doses (Figure 5A). Two weeks post‐inoculation, the infected mice were divided into three treatment groups: 0.5% CMC‐Na (control), a combination of amoxicillin, clarithromycin, and omeprazole (OPZ+AC), or a combination of CA and omeprazole (OPZ+CA). The therapeutic efficacy was determined by quantifying bacterial loads in the stomachs. As shown in Figure 5B, the dual therapy of CA and omeprazole achieved bacterial clearance comparable to the standard triple therapy, reducing the bacterial burden by approximately 1000‐fold relative to the control.

Because NSH57 strain is antibiotic‐sensitive, this mouse model could not fully recapitulate clinical drug resistance. We therefore utilized a second model using a mouse‐adapted strain BHKS00388 [56], derived from a human clinical isolate resistant to levofloxacin, clarithromycin, and amoxicillin. This allowed us to assess the in vivo efficacy of CA against multidrug‐resistant *H. pylori*. Following a 4‐day treatment regimen, the OPZ+CA group exhibited an approximately 4‐log reduction in bacterial burden compared to the control. Notably, the dual therapy significantly reduced colony counts and outperformed the triple therapy group (Figure 5C).

Finally, to evaluate the potential toxicity of CA, we performed histopathological analysis on longitudinal sections of gastric tissue stained with hematoxylin and eosin (H&E) (Figure 5D). Gastric sections from *H. pylori*‐infected mice treated with the control (CMC‐Na) showed marked inflammation, characterized by ulcer craters and heavy immune cell infiltration into the muscularis mucosae. In contrast, the gastric architecture of mice treated with OPZ+CA remained intact, with a well‐defined epithelial cell layer resembling that of the uninfected control (blank). Furthermore, CA treatment did not increase gastric epithelial apoptosis, as assessed by TUNEL assay. No significant changes in body weight were also observed in any infected group compared to the uninfected control (blank) over the course of the experiment (Figure 5E). In addition to gastric histopathology, the systemic safety of CA was further evaluated through an acute toxicity assessment in both female and male mice following a single oral dose of 2000 mg kg^−1^ CA. CA administration produced no overt changes in body weight in either sex, exhibited no detectable hemolytic activity, and did not significantly alter hematological or serum biochemical parameters associated with liver and kidney function (Figure S5). Collectively, these results indicate that CA is a highly effective and safe lead compound against *H. pylori* infection in vivo, including strains that are multidrug‐resistant.

### 2.6 CA Causes Minor Changes to Gut Microbiota

**FIGURE 6:**
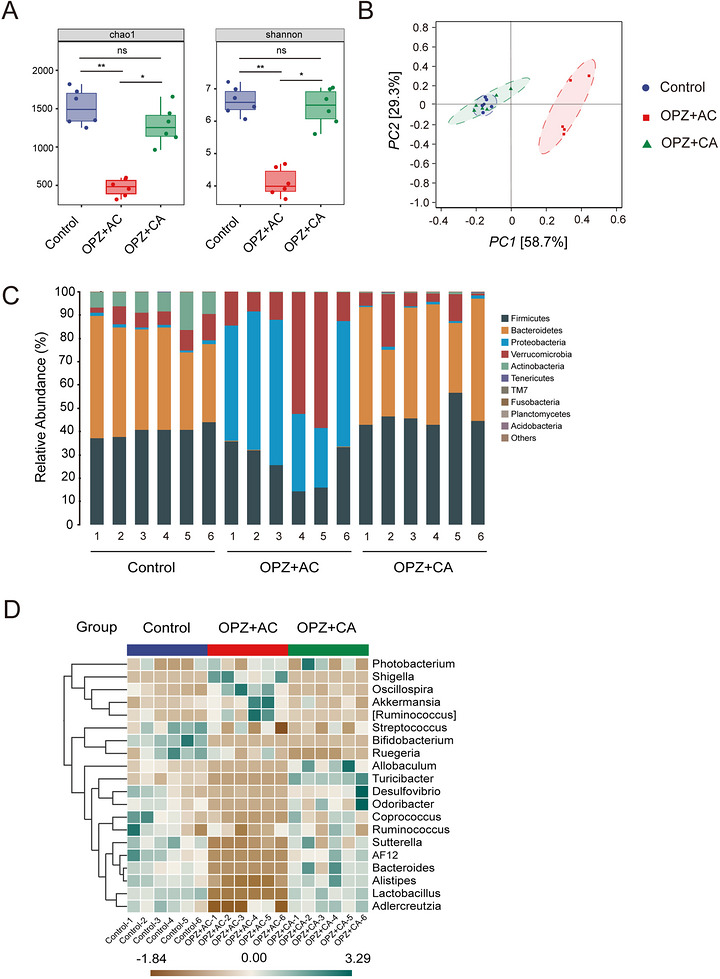
CA induces minimal changes in the diversity and composition of the fecal bacterial flora. (A) ɑ‐diversity, calculated from 16S rRNA gene sequencing data using the Chao1 (left panel) and Shannon (right panel) indices, was assessed for the three groups treated with CMC‐Na (control), OPZ + CA, and triple therapy (OPZ + AC). Statistical differences between the control and treatment groups were analyzed using the Kruskal‐Wallis test followed by Dunn's multiple comparisons test. *, *p* < 0.05; **, *p* < 0.01. Box‐and‐whisker plot shows the median (line), interquartile range (box), and minimum–maximum values (whiskers). (B) Principal component analysis (PCA) of the three groups was based on operational taxonomic unit (OTU) abundance, with the percentage contribution of each principal component indicated in parentheses. Each dot represents an individual sample. (C) Relative abundances of bacterial phyla identified in each sample from the sequencing data. Data were clustered by sample group along the x‐axis. (D) Heat map comparing the average abundances of bacterial genera in 16S rRNA sequences from each sample under different treatments.

To characterize the impact of CA on the gut microbiota, we collected stool samples from each group and performed 16S rRNA gene (V3–V4 region) sequencing. Analysis of alpha‐diversity, measured by the Chao1 (estimating operational taxonomic unit [OTU] richness) and Shannon (assessing richness and evenness) indices, revealed no significant differences between the control and CA dual‐therapy groups (Figure 6A). In contrast, a significant reduction in alpha‐diversity was observed following triple therapy. Beta‐diversity was assessed using principal component analysis (PCA), which showed a distinct separation of the triple therapy group along the first principal component (PC1), accounting for 58.7% of the variation and indicating a substantial shift in the core microbiota (Figure 6B). The microbiota profile of the OPZ+CA group, however, remained similar to that of the control. This was further supported by weighted UniFrac distance analysis, which confirmed minimal dissimilarity in microbial species and abundance between the OPZ+CA and control groups (Figure S6A). Collectively, these α‐ and β‐diversity analyses demonstrate that CA treatment preserves the native gut microbiota structure, whereas triple therapy induces significant dysbiosis.

Next, we compared the composition of the microbiota to identify differences in bacterial taxa among the groups. At the phylum level, Bacteroidetes and Firmicutes predominated in both the control and CA dual‐therapy groups (Figure 6C). The triple‐therapy group, however, exhibited a marked expansion of *Proteobacteria* and *Verrucomicrobiota*, which together accounted for nearly 50% of the total abundance. Genus‐level analysis revealed a decline in beneficial genera such as *Lactobacillus*, *Bacteroides*, and *Alistipes* in the triple‐therapy group, alongside a significant increase in Shigella (Figure 6D). In contrast, the OPZ+CA and control groups maintained similar compositional profiles, as also observed at the family level (Figure S6B). Overall, these findings suggest that, compared to triple‐therapy, CA treatment induces only minor changes in the diversity and composition of the murine gut microbiota.

## 3 Discussion and Conclusions

The efficacy of conventional triple or quadruple therapies for *H. pylori* infection is increasingly compromised by the rise of antibiotic resistance [12]. Furthermore, these broad‐spectrum antibiotics frequently disrupt the ecological balance of the gut microbiota, creating an urgent need for novel agents that can selectively target *H. pylori*. In this study, we demonstrate that the TCM monomer carnosic acid exhibits potent and specific activity against *H. pylori* both in vitro and in vivo. Notably, a combination therapy of CA and a PPI proved more effective than standard triple therapy in a mouse model of multidrug‐resistant *H. pylori* infection. Crucially, CA treatment did not significantly alter the composition or diversity of the gut microbiota in mice, underscoring its potential as a promising narrow‐spectrum lead compound.

Mechanistically, our findings reveal that CA employs a multi‐pronged strategy against *H. pylori*. It significantly impairs bacterial motility, suppresses urease activity through direct binding to the UreA subunit, and disrupts cell membrane integrity and function. Among these effects, disruption of the bacterial cell membrane is likely the primary lethal event, as loss of membrane integrity directly compromises bacterial viability and leads to irreversible cell death. This coordinated attack, which merges direct bactericidal action with the suppression of key virulence traits, represents a promising strategic avenue for combating resilient bacterial pathogens. The multi‐mechanistic nature of CA provides a distinct advantage over conventional single‐target antibiotics, such as clarithromycin, levofloxacin, and metronidazole, whose efficacy is increasingly compromised by resistance. Notably, while membrane disruption drives bactericidal activity, inhibition of urease and motility may predominantly affect earlier stages of infection by impairing acid resistance and gastric colonization, and antibiofilm activity targets a distinct physiological state associated with persistence and tolerance. By simultaneously targeting membrane integrity, biofilm formation, motility, and urease‐mediated acid adaptation, CA acts across multiple stages of pathogenesis, thereby enhancing bactericidal efficacy and potentially circumventing existing resistance mechanisms.

The propensity for resistance development is a critical factor in selecting anti‐*H. pylori* drugs. Remarkably, we observed no induced resistance in *H. pylori* after 60 days of serial passaging in sub‐inhibitory concentrations of CA. Furthermore, no spontaneous CA‐resistant mutants were isolated in multiple independent selection experiments. This extremely low resistance frequency is likely attributable to CA's multi‐targeting mechanism, wherein concurrent alterations in several essential functions would be required for resistance to emerge. Additionally, the ultralow prevalence of CA resistance suggests that any potential resistant mutations may incur a high fitness cost, further suppressing their selection.

A key feature of CA is its narrow‐spectrum antibacterial activity, which is crucial for preserving gastrointestinal homeostasis. Unlike broad‐spectrum antibiotics that often cause dysbiosis and secondary infections [57], CA's specificity for *H. pylori* suggests a lower risk of disturbing commensal microbiota and reduced potential for systemic toxicity. The specificity of CA may be attributed to certain physiological or structural features that distinguish *H. pylori* from non‐target species. This characteristic aligns with the emerging paradigm of precision antimicrobial therapy [58], which aims to selectively eliminate pathogens while minimizing unintended impacts on the host microbiome.

As an oral therapeutic candidate, the “druggability” of CA is a major concern that is currently unaddressed. Although absorption and metabolic behavior were not directly evaluated in the present in vivo model, previous pharmacokinetic studies have confirmed that orally administered CA is detectable in rat stomach and intestinal tissues, as well as in plasma. Tissue distribution analysis revealed a relatively high concentration of CA in the stomach wall, which gradually decreased over time, with a maximum concentration of 307.1 ± 119.2 µg g^−1^ following oral administration of CA (90 mg·kg^−^^1^) [59, 60]. Notably, purified CA has been reported to remain chemically stable under acidic conditions (pH 2) for at least 2 h, supporting its intrinsic stability in a gastric‐like environment [61]. Comprehensive ADME studies further demonstrate that CA is absorbed, enters systemic circulation, and exhibits defined distribution and elimination profiles in vivo, with these pharmacokinetic behaviors being influenced by its lipophilic phenolic diterpene structure [62, 63]. Moreover, toxicological evaluations have shown that CA possesses relatively low acute toxicity (LD_50_ ≈ 7100 mg kg^−^^1^ in Kuming mice) and elicits only minor effects upon repeated oral administration [64]. Consistently, in our acute toxicity evaluation, a single oral dose of 2000 mg kg^−^^1^ CA did not result in significant body weight changes or observable adverse effects. It is worth noting that CA exhibited some cytotoxicity toward gastric cancer cell lines, with IC_50_ values of approximately 20 µg mL^−^^1^ [65]. Given the anti‑*H. pylori* MIC_50_ of 8 µg mL^−^^1^, the calculated selectivity index (SI) is approximately 2.5. We acknowledge that this relatively narrow in vitro SI warrants caution. Future preclinical studies should therefore evaluate CA's safety profile in normal gastric epithelial cells and assess long‐term chronic dosing regimens before clinical translation, in order to minimize any potential off‐target effects.

Beyond its direct antibacterial activity, the chemical tractability of CA further supports its potential as a lead scaffold for anti‐*H. pylori* drug development. CA belongs to the abietane diterpenoid family, a class of natural products that has attracted increasing interest in medicinal chemistry due to their tunable physicochemical properties and membrane‐active profiles. Recent work has demonstrated that rational modification of the abietane diterpenoid scaffold can substantially enhance antibacterial potency, broaden activity spectra, and improve pharmacokinetic behavior, while preserving the core bioactive framework [66]. In parallel, semisynthetic derivatization of CA and closely related compounds has been shown to markedly alter biological activity through targeted modification of peripheral functional groups, underscoring the sensitivity of this scaffold to chemical optimization [67]. These studies suggest that CA represents not only a bioactive natural product but also a chemically evolvable starting point for further lead optimization aimed at improving gastric exposure, selectivity, and translational potential.

In conclusion, our findings establish CA as a potent, specific, and microbiota‐friendly anti‐*H. pylori* agent with a favorable preliminary safety profile. Its unique multi‐mechanistic action, which includes inhibiting motility and urease activity while disrupting membrane integrity, distinguishes it from conventional antibiotics and minimizes the risk of cross‐resistance. These compelling attributes position CA as a valuable lead compound for developing precision therapeutics against *H. pylori* infection.

## 4 Experimental Section

### 4.1 Chemicals and Antimicrobial Agents

Carnosic acid (CAS no. 3650‐09‐7, purity ≥ 98.7%), carnosol (CAS no. 5957‐80‐2, purity ≥ 99.3%), rosmanol (CAS no. 80225‐53‐2, purity ≥ 99.0%), salviolone (CAS no. 119400‐86‐1, purity ≥ 99.3%), and 20‐Deoxocarnosol (CAS no. 94529‐97‐2, purity ≥ 98.0%) were purchased from MedChemExpress (Monmouth Junction, NJ, USA). Four antibiotics (amoxicillin, purity ≥ 98.0%, metronidazole, purity ≥ 99.0%, clarithromycin, purity ≥ 98.0%, and levofloxacin, purity ≥ 98.0%) and omeprazole (commercially available tablets, each containing 20 mg active ingredient) were obtained from Aladdin Reagent (Shanghai) Co. Ltd. (Shanghai, China). Other chemicals (purity ≥ 98.0%) were obtained from Sigma–Aldrich (St. Louis, MO, USA) or Sinopharm Chemical Reagent Co. Ltd. (Shanghai, China). Stock solutions of these chemicals were prepared in dimethyl sulfoxide (DMSO).

### 4.2 Bacterial Strains and Culture Conditions

*H. pylori* strains G27, 26695, NSH57 and 38 clinical isolates (including 5 freshly isolated clinical strains) used in this study were routinely cultured in either brain heart infusion (BHI) broth (Becton, Dickinson, Sparks, MD, USA) medium containing 10% fetal calf serum (FCS) or Columbia blood agar (Oxoid, Basingstoke, UK) plates containing 10% FCS. All plates and media were incubated at 37°C for 48–72 h under microaerophilic conditions (10% CO_2_, 85% N_2_, and 5% O_2_ at 90% relative humidity) using a tri‐gas CO_2_ incubator (model CB160; Binder, Germany). A total of 38 local strains of *H. pylori* were isolated from biopsy samples of patients with gastritis or gastric cancer using standard protocols. The specimens were obtained from Sir Run Run Hospital and the First Affiliated Hospital of Nanjing Medical University, China. The strains were identified based on colony appearance, Gram staining, and positive reactions with the rapid urease test. Non‐*Helicobacter* bacterial and fungal strains were cultured as described previously [68].

### 4.3 MIC Assays

The MICs against *H. pylori* were determined using a broth microdilution assay as described previously [68]. Twofold serial dilutions of the test compounds were prepared in 96‐well microtiter plates containing 100 µL of BHI broth supplemented with 10% FCS. An overnight *H. pylori* culture was diluted in BHI broth and inoculated into each well to achieve a final concentration of approximately 5 × 10^5^ CFU mL^−1^. The plates were incubated for 48 h at 37°C in a microaerophilic atmosphere. After incubation, the plates were inspected visually, and the MIC was defined as the lowest concentration at which no turbidity was observed. The MICs against other bacterial strains and fungal strains were determined as described previously [68]. All MIC assays were repeated with at least three independent experiments.

### 4.4 Time‐Kill Curves

An overnight liquid culture of *H. pylori* G27 was grown to the exponential phase with shaking at 200 rpm. The culture was then diluted in BHI broth supplemented with 10% FCS to a concentration of 1 × 10^6^ CFU mL^−1^. Various concentrations of CA and MTZ (1×, 2×, 4×, and 8× MIC) or an equivalent amount of DMSO (vehicle control) were added. The cultures were incubated at 37°C in a microaerophilic atmosphere with shaking at 200 rpm. Aliquots were removed at specified time points over 48 h, serially diluted, and plated for CFU enumeration. Bactericidal activity was assessed by the reduction in viable counts.

### 4.5 Drug Resistance Study

For single‐step resistance, 10^10^ CFU of *H. pylori* G27 were plated onto Columbia blood agar base containing 2×, 4×, or 8×MIC of CA. After one week of incubation at 37°C, no resistant colonies were detected. The development of drug resistance by sequential passaging was investigated using a previously reported method [69]. This was assessed by serially passaging *H. pylori* G27 in sub‐inhibitory concentrations of CA and MTZ for up to 15 cycles (60 days). The MIC values after each cycle of continued exposure were determined using the broth microdilution method described above.

### 4.6 Anti‐Biofilm Assay

The effects of CA on biofilm formation and on preformed biofilms were evaluated using *H. pylori* G27 as previously described [69]. Overnight cultures grown in Brucella broth supplemented with 10% FBS were diluted to an OD_600_ of 0.15 and inoculated into triplicate wells of 96‐well polystyrene plates (200 µL per well). For biofilm inhibition assays, varying concentrations of CA or MTZ (positive control), or an equivalent volume of DMSO, were added simultaneously and incubated at 37°C under microaerophilic conditions for 48 h. For biofilm eradication assays, biofilms were allowed to form for 72 h, followed by replacement with fresh medium containing CA or MTZ and further incubation for 24 h. Biofilm biomass was quantified by crystal violet staining [70]. Data are presented as mean ± s.d. from three independent experiments.

### 4.7 Quantifying the Effect of CA on Bacterial Viability in Biofilms

The effects of CA and MTZ on preformed biofilm viability were assessed by CFU counting as previously described [69]. *H. pylori* G27 cells were cultured in triplicate in 96‐well plates under microaerobic conditions for 72 h. Wells were washed with PBS, treated with 200 µL of 1% saponin, and biofilms were scraped and pipetted 15 times for complete disruption. Suspensions were serially diluted, plated, and viable colonies were counted as CFU/mL.

### 4.8 Biofilm Analysis by Confocal Microscope

Biofilm viability was evaluated using the Live/Dead BacLight Bacterial Viability Kit (Invitrogen, Molecular Probes, USA), containing the fluorescent dyes SYTO9 and PI as previously described [69]. *H. pylori* G27 biofilms were grown in orifice plates for 72 h, then treated with CA, MTZ, or DMSO for 24 h. After three PBS washes, biofilms were stained with SYTO9/PI for 30 min at 37°C in the dark. Images were acquired using a confocal microscope (LSM710; Carl Zeiss, Oberkochen, Germany). SYTO9 stains total cells (green), and PI stains membrane‐damaged cells (red). Quantitative analysis was performed using ImageJ software by calculating the proportion of green‐stained cells relative to the total number of cells (green + red) in each image.

### 4.9 Biofilm Analysis by Alamar Blue Staining Methods

*H. pylori* G27 cells were prepared and treated as described above for preformed biofilm eradication. After 24 h incubation with CA or MTZ, 20 µL of Alamar Blue (Solarbio; Beijing, China) was added to each well and incubated for an additional 3 h. Cell viability was assessed by visual inspection of the color change in the medium, as viable cells reduce Alamar Blue to a fluorescent pink color, whereas nonviable cells do not.

### 4.10 Soft‐Agar Motility Assay

Bacterial motility was assessed using a modified soft‐agar assay, as previously described [71]. An overnight liquid culture of *H. pylori* G27 was grown to the exponential phase with shaking at 200 rpm. The culture was then diluted 1:5 in Brucella broth supplemented with 10% FCS and treated with the specified concentrations of each compound for 8–12 h at 37°C under microaerophilic environments. A 3 µL aliquot of the bacterial suspension (OD_600_ = 3.0) was then inoculated as a central drop onto soft Brucella agar plates (0.35% agar) containing 5% FBS (v/v) and the corresponding compound concentrations. Plates were incubated at 37°C for 5 days under microaerophilic conditions. Motility was quantified by measuring the diameter of bacterial migration from the inoculation point to the colony edge. Results were normalized to the diameter of total bacterial growth for each condition. Data are presented as the mean ± standard deviation from three independent experiments.

### 4.11 Assessment of Bacterial Motility via Dark‐Field Microscopy

An overnight liquid culture of *H. pylori* G27 was grown to exponential phase and diluted in BHI broth to an OD_600_ of 0.3. The test compounds, CA and the reference drug MTZ, were individually added to separate cultures at a final concentration of 1/2× MIC, followed by a 6‐h incubation period. Bacterial motility was observed under a dark‐field microscope (Nikon Ci‐s, Japan). Video sequences were captured at a rate of one frame every 20 ms for a total of 500 frames per sample. Individual bacterial trajectories were analyzed using the Channel and Spatial Reliability‐Aware Tracker (CSRATracker) algorithm implemented in Python. This algorithm was used to track single bacterial cells and calculate associated motion parameters, including speed and acceleration.

### 4.12 Qualitative Detection of Inhibition Against *H. pylori* Urease

The inhibitory activity of CA against *H. pylori* urease was visually assessed using Christensen's urea broth [72]. Each liter of the medium contained 1 g peptone, 5 g NaCl, 1 g glucose, 2 g KH_2_PO_4_, 1.2 g Na_2_HPO_4_, and 0.012 g phenol red, with the pH adjusted to 6.8–7.4. The autoclaved Christensen medium, supplemented with 2% urea, was prepared in a 96‐well microtiter plate. An overnight *H. pylori* G27 culture was diluted and inoculated into each well to achieve a final concentration of 1 × 10^7^ CFU mL^−1^. Different concentrations of CA were added to each well using a multichannel pipette. After 6 h of incubation, absorbance values at OD_570_ were measured using a microplate reader. AHA (Sigma–Aldrich) served as the positive control, while DMSO was used as the negative control.

### 4.13 Cloning, Expression, and Purification

The *ureA* gene was amplified from the genomic DNA of *H. pylori* strain G27 by the polymerase chain reaction (PCR) and cloned into the expression vector pQE‐2 (Qiagen), which encodes UreA with an N‐terminal 6× hexahistidine (His) tag. UreA was expressed in Rosetta (DE3) pLysS cells, which were grown at 37°C in LB medium. When the OD_600_ reached 0.6–0.8, the cultures were induced with 0.2 mM isopropyl‐β‐D‐thiogalactoside (IPTG) and incubated at room temperature for 12 h before harvest. The cells were collected, resuspended in lysis buffer (50 mM NaH_2_PO_4_, 300 mM NaCl, 10 mM imidazole, 1 mM β‐mercaptoethanol, pH 8.0), lysed by ultrasonication, and centrifuged at 12 000 rpm for 60 min. The clarified bacterial supernatant was applied to a nickel‐ion affinity column (Qiagen). The column was washed with wash buffer (50 mM NaH_2_PO_4_, 300 mM NaCl, 40 mM imidazole, 1 mM β‐mercaptoethanol, pH 8.0) to remove contaminating proteins, and His‐tagged UreA protein was eluted with elution buffer containing 200 mM imidazole. The protein was concentrated using ultrafiltration with a 10 kDa cut‐off and exchanged into sodium phosphate buffer (50 mM NaH_2_PO_4_, 300 mM NaCl, 1 mM β‐mercaptoethanol, pH 8.0). Protein purity was assessed by gradient SDS‐PAGE (12%).

### 4.14 Extraction of *H. pylori* Urease

The urease of *H. pylori* was extracted using a previously reported method [73]. *H. pylori* strain G27 was cultured under microaerobic conditions in Brucella broth supplemented with 10% FCS overnight. The bacterial culture was then expanded into 200 mL of the same medium at a 1:100 dilution and incubated for an additional 24 h. The culture was centrifuged at 4700 rpm for 15 min at 4°C and washed twice with PBS. The resulting precipitate was stored at −80°C for 24 h, then thawed and brought to room temperature. The bacteria were resuspended in an appropriate volume of dialysis buffer (50 mM NaH_2_PO_4_, 300 mM NaCl, 1 mM β‐mercaptoethanol, pH 7.0) and supplemented with 1 mM PMSF. After ultrasonic fragmentation, the mixture was centrifuged at 12 000 rpm for 30 min. The bacterial pellet was discarded, glycerol was added to the clarified bacterial supernatant to a final concentration of 50%, and the mixture was stored at −80°C.

### 4.15 Measurement of Urease Inhibitory Activity

Urease activity was assessed using a 96‐well plate by measuring ammonia production through the indophenol method with minor modifications [74]. Briefly, 50 µL of urease solution in dialysis buffer at the appropriate concentration was incubated with various concentrations of compounds at 37°C for 20 min. Following incubation, 50 µL of 100 mM urea solution was added to each well, mixed thoroughly, and left to react at room temperature in the dark for 20 min. Next, 50 µL of Berthelot solution A (containing 9.73 mM sodium nitroprusside and 700 mM sodium salicylate) and 50 µL of Berthelot solution B (containing 4.5 M NaOH and 35 mL NaClO in 100 mL) were added and mixed. The mixture was incubated at room temperature for an additional 10 min to develop color. Absorbance was measured at 635 nm using a microplate reader (BioTek Instruments, Winooski, VT, USA), with AHA as a reference. Percentage inhibition was calculated, and experiments were performed in triplicate. The IC_50_ value was defined as the concentration of the compound causing 50% inhibition of maximal activity.

### 4.16 Enzymatic Characterization

Kinetic analysis was performed to determine the mode of inhibition. Kinetics were assessed using various urea concentrations (10, 5, 2.5, 1.25, and 0.625 mM) in the presence of different concentrations of CA (2.4, 9.4, and 37.6 µM). The remaining procedures followed the previously mentioned protocol for measuring urease inhibitory activity. Results, represented as changes in absorbance per minute, were recorded at 635 nm with a microplate reader. The enzyme inhibition type was determined using Lineweaver–Burk plots, which display the inverse of velocities (1/V) versus the inverse of substrate concentration (1/[S] mM^−1^). Data fitting was performed using the mixed inhibition model in GraphPad Prism 8.0 software.

### 4.17 Silver Staining Experiment

Silver staining was performed following a previously established protocol [75]. CA at final concentrations of 100, 500, and 2500 µg mL^−1^, or DMSO (used as a negative control), was co‐incubated with HpUreA or BSA at room temperature for 1 h. Subsequently, 1.25 µg of *Streptomyces* pronase was added to each sample. After an additional 30 min of incubation, the samples were analyzed using gradient SDS‐PAGE (18%).

### 4.18 Microscale Thermophoresis (MST) Analysis

MST experiments were conducted using a Monolith NT.115 instrument (NanoTemper, Germany) at 20% LED power, following a previously described method with minor modifications [76]. HpUreA was labeled with a RED‐NHS dye kit (NanoTemper Technologies) and adjusted to a concentration of 25 nM in PBST. The ligand CA was dissolved in PBST, and a series of 16 (1:1) dilutions were prepared, resulting in ligand concentrations ranging from 0.48 nM to 16 µM. For measurement, each ligand dilution was mixed with an equal volume of labeled HpUreA, resulting in ligand concentrations ranging from 0.24 nM to 8 µM. The samples were loaded into Monolith NT.115 capillaries, which measured the change in extrinsic fluorescence of HpUreA upon ligand binding. Fluorescence data were fitted to the MST curve using the “*K*_d_ Model” in MO Affinity Analysis software (NanoTemper, Germany) and normalized to the fraction bound (ranging from 0 to 1) for each data point by dividing by the curve amplitude.

### 4.19 Docking Study

To probe the CA interaction with the potential binding sites of *H. pylori* UreA, molecular docking between them was carried out using Schrödinger 2015 software. The structure of the *H. pylori* urease (PDB Code: 1E9Y) was downloaded from the RCSB Protein Data Bank (https://www.rcsb.org/structure/1E9Y). The 3D structures of CA (PubChem CID: 65126) were retrieved from the NCBI PubChem database (https://pubchem.ncbi.nlm.nih.gov/compound/65126).

### 4.20 TEM

The effects of CA on *H. pylori* structure were examined using TEM using a previously published procedure [56]. Briefly, an overnight culture of *H. pylori* G27 at a concentration of 10^8^ CFU mL^−1^ was treated with CA (1×MIC and 2×MIC) and incubated at 37°C for 1 h under microaerobic conditions. Polymyxin B (PMB, MDBio, Shandong, China) served as the positive control, while DMSO was used as the negative control. Samples were centrifuged at 2000 g for 10 min, then washed once with fetal calf serum (FCS) and once with phosphate‐buffered saline (PBS). Bacterial pellets were fixed by resuspension in 2% glutaraldehyde in 0.1 M sodium cacodylate buffer (pH 7.4). The bacterial pellets were then embedded in 2% agarose and post‐fixed with 1% osmium tetroxide overnight at room temperature. After washing, the samples were dehydrated in increasing concentrations of ethanol and embedded in Durcupan resin (Sigma–Aldrich). Fifty‐five‐nanometer sections were examined using a JEM‐1200 transmission electron microscope (JEOL, Akishima, Tokyo, Japan) equipped with a 4K Eagle digital camera (FEI, Hillsboro, OR, USA). In addition, *E. coli* ATCC25922 and *P. aeruginosa* PAO1 were treated with DMSO, CA (128 µg mL^−^^1^), or PMB (5 µg mL^−^^1^) for 4 h, followed by TEM analysis to assess membrane integrity.

### 4.21 Outer Membrane Permeability Assay

Outer membrane permeability was assessed using the fluorescent probe N‐phenyl‐1‐naphthylamine (NPN). Overnight cultures of *H. pylori* G27 were collected, resuspended in BHI medium supplemented with 10% FCS, and adjusted to an OD_600_ of 0.5. CA was added to final concentrations of 1× MIC (16 µg mL^−^^1^) and 2× MIC (32 µg mL^−^^1^), with PMB as a positive control. After incubation for 1 h under microaerobic conditions, NPN was added to a final concentration of 10 µM and incubated for an additional 20 min. Fluorescence was measured using a multimode microplate reader (excitation 360 nm; emission 440 nm).

### 4.22 SEM

The surface morphology of *H. pylori* was examined by scanning electron microscopy (SEM). Briefly, *H. pylori* G27 (1 × 10^8^ CFU mL^−^^1^) was treated with DMSO, CA (1× and 2× MIC), or PMB (1× and 2× MIC) for 1 h. Cells were gently washed with PBS, collected by centrifugation, and fixed with electron microscopy fixative (Wuhan Servicebio Technology) for 30 min at room temperature in the dark. Samples were washed three times with PBS for 15 min each, post‐fixed in 1% osmium tetroxide for 1 h, and washed again with PBS. Samples were dehydrated through a graded ethanol series (30–100%) followed by isoamyl acetate, and then subjected to critical point drying. The dried samples were mounted on conductive carbon tape, sputter‐coated with gold for 30 s, and observed using a SEM (Hitachi SU8100). Images were acquired for analysis.

### 4.23 Membrane Integrity Assays

An evaluation of cell membrane integrity was performed according to a previously reported method [77]. Overnight cultures of *H. pylori* G27 were centrifuged and resuspended in BHI medium containing 10% FCS to achieve a bacterial suspension with an OD_600_ of 0.5. CA was then added to final concentrations of 1×MIC (16 µg ml^−1^) and 2×MIC (32 µg ml^−1^), with PMB serving as a positive control. After incubating for 1 h under microaerobic conditions, the bacterial suspensions were treated with PI at a final concentration of 10 nM and incubated for an additional 20 min. Fluorescence was measured using a Multimode Microplate Reader (Cytation 5; Biotek, USA) with excitation and emission wavelengths of 535 and 615 nm, respectively. For protein concentration measurement, 1 mL of the bacterial suspension was centrifuged at 12 000 rpm for 2 min after 4 h of compound treatment. The bacterial pellet was resuspended in 50 µL of lysis solution (Beyotime Biotechnology; Shanghai, China). Protein concentration was determined using the Bradford method with Bradford reagent (Sigma–Aldrich). In addition, *E. coli* ATCC25922 and *P. aeruginosa* PAO1 were treated with DMSO, CA (128 µg mL^−^^1^), or PMB (5 µg mL^−^^1^) for 1 h, followed by PI staining under the same conditions to assess membrane permeability. All assays were performed in at least triplicate.

### 4.24 ATP Determination

The release of ATP from cells was quantified as an indicator of plasma membrane permeability. Briefly, approximately 3 × 10^7^ *H. pylori* G27 cells were treated with PMB, CCCP, or CA (at 1×MIC and 2×MIC) for 1 h, with DMSO serving as a negative control. Following treatment, the OD_600_ of each suspension was recorded. The samples were then centrifuged, and the supernatant was carefully separated from the cell pellet. The pellet was resuspended in 200 µL of PBS. Subsequently, 100 µL aliquots of both the supernatant and the resuspended pellet were transferred to an OptiPlate‐96F solid‐bottom white plate. An equal volume (100 µL) of BacTiter‐Glo reagent (Promega Corporation) was added to each well, and luminescence was measured immediately using a microplate reader. The ATP concentration in each sample was determined by interpolation from a standard curve. All assays were performed in at least triplicate.

### 4.25 Membrane Potential Assay

Inner membrane depolarization was assessed using the membrane potential (Δψ)‐sensitive fluorescent probe 3,3‐Diethylthiacarbocyanine iodide (DiSC_2_(3); MedChemExpress) as described previously [78]. Briefly, overnight cultures of *H. pylori* G27 were washed and resuspended in PBS to an OD_600_ of 0.3. A 5 mL aliquot of the bacterial suspension was incubated with DiSC_2_(3) at a final concentration of 1 µM, and the dye was allowed to equilibrate. The suspension was then dispensed into a 96‐well black microplate. Fluorescence was recorded for 10 min to establish a stable baseline using a multimode microplate reader (excitation 488 nm; emission 620 nm). Subsequently, test compounds (DMSO, CA at 32 µg mL^−1^, or CCCP at 0.25 µg mL^−1^) were added, and fluorescence was continuously monitored for an additional 60 min to assess changes in membrane potential.

### 4.26 ΔpH Measurement

Overnight cultures of the *H. pylori* G27 strain were centrifuged, washed with HEPES buffer (5 mM, pH 7.0, plus 5 mM glucose), and resuspended to an OD_600_ of 0.5. The ΔpH was measured using the pH‐sensitive fluorescence probe BCECF‐AM (Beyotime Biotechnology, China) [79]. BCECF‐AM was added to a final concentration of 5 µM and incubated at 37°C for 20 min. Fluorescence was measured for the first 10 min. Subsequently, 1 µL of CA or CCCP solution (final concentration of 32 µg mL^−1^) was added, and fluorescence was recorded for an additional 30 min using a Multimode Microplate Reader (Cytation 5; Biotek, USA), with excitation at 488 nm and emission at 535 nm.

### 4.27 ROS Measurement

The ROS generated by CA was measured using the fluorescence probe 6‐chloromethyl‐2',7'‐dichlorodihydrofluorescein diacetate, acetyl ester (CM‐H_2_DCFDA; Genmed Scientifics, Inc.). Briefly, *H. pylori* G27, cultured overnight, was resuspended to an OD_600_ of 0.1. CA or amoxicillin at 1×MIC was added to the bacterial suspension and incubated under microaerobic conditions at 37°C for 4 h. A 100 µL aliquot of the treated bacterial suspension was transferred to a 96‐well black microplate and mixed with 2.5 µL of the fluorescence probe. After an additional 30 min of incubation, fluorescence values were measured using a Multimode Microplate Reader (Cytation 5; Biotek, USA) with an excitation wavelength of 490 nm and an emission wavelength of 530 nm. All assays were performed in at least triplicate.

### 4.28 Efficacy of CA against *H. pylori* In Vivo

Mouse‐adapted *H. pylori* strains NSH57 and BHKS00388 (domesticated from a human clinical BYES00388 strain with resistance to metronidazole, clarithromycin, and amoxicillin) [56, 68], were used for infection. Mice were sourced from the Animal Core Facility at Nanjing Medical University, China, and housed at the same place in a 12‐h light–dark cycle with ambient temperature and humidity maintained at 68–74°F and 30–70%, respectively. All experimental procedures were performed in a biosafety level two laminar flow hood. All animals were healthy upon receipt and were weighed and monitored during a 7‐day acclimation period before the start of the study.

Six‐week‐old specific‐pathogen‐free female C57BL/6 mice received 0.3 mL of *H. pylori* suspension at a concentration of 1 × 10^9^ CFU mL^−1^ in BHI broth via oral gavage every 48 h for a total of five administrations. The infection was allowed to develop for 2 weeks. The mice were then randomly assigned to three groups (n = 8) and treated as follows: CA plus omeprazole, triple therapy, or a solvent (0.5% CMC‐Na and 0.2% Tween 80). Omeprazole (400 µmol kg^−1^ day^−1^) was administered by oral gavage 30 min before the assigned treatments. CA (30 mg kg^−1^ day^−1^) and the triple therapy formulation (amoxicillin at 30 mg kg^−1^ day^−1^ and clarithromycin at 15 mg kg^−1^ day^−1^) were administered once daily for four consecutive days by oral gavage. The control group received an equivalent volume of the solvent. Two days after the final treatment, fecal samples were collected from the control and treated mice, which were then humanely euthanized by gradual‐fill CO_2_ inhalation in accordance with the guidelines of the Institutional Animal Care and Use Committee (IACUC, approval no. 2005036), with CO_2_ flow introduced at a displacement rate of 20–30% of the chamber volume per minute to minimize distress, and their stomachs were harvested. Each stomach was cut along the greater curvature into two longitudinal sections, and each section was weighed after removal of the gastric contents. The sections were used for assessments of bacterial colonization, H&E staining, and TUNEL staining. For bacterial colonization, the gastric tissue was suspended in 1 mL of BHI broth supplemented with 10% FCS and gently homogenized for *H. pylori* recovery. The suspensions were serially diluted, streaked onto Columbia blood agar plates containing selective antibiotics and 10 µg mL^−1^ bacitracin, and incubated at 37°C under microaerobic conditions for five days. Viable bacterial colonies were counted and expressed as CFU per gram of stomach tissue. H&E staining and TUNEL staining were performed by Servicebio Biotechnology Co., Ltd (China).

### 4.29 Acute Toxicity Study

Acute toxicity was evaluated in ICR mice of similar body weight. Animals were randomly divided into two groups (n = 6 per group; 3 males and 3 females), including a vehicle control and a CA‐treated group (2000 mg kg^−^^1^). Body weight was recorded daily for 14 days following a single gavage administration. At the end of the observation period, blood samples were collected for hematological and biochemical analyses, which were performed by Jiangsu Aidisheng Biological Technology Co.,Ltd (China).

### 4.30 Bacterial Community Composition and Diversity

The fecal bacterial community composition was analyzed by partial 16S rRNA gene sequencing of the extracted community DNA. The V4 hypervariable regions of the 16S rRNA gene were amplified using primers F (5’‐ACTCCTACGGGAGGCAGCA‐3’) and R (5’‐GGACTACHVGGGTWTCTAAT‐3’), as previously described [56]. The PCR products were purified using the OMEGA Soil DNA Kit (M5635‐02) (Omega Bio‐Tek; Norcross, GA, USA). Purified amplicons from 18 samples, divided into three groups (6 samples per group) were sequenced on an Illumina MiSeq PE250 sequencer at Personalbio Biotechnology Co., Ltd. (Shanghai, China). Following sequencing, the Illumina Analysis Pipeline version 2.6 was used for image analysis, base calling, and error estimation. Qualified reads from the 18 raw data samples were clustered into operational taxonomic units (OTUs) at a 97% similarity threshold using the Uparse algorithm in Vsearch software (v.2.13.4) [80]. The National Center for Biotechnology Information (NCBI) 16S rRNA sequence and taxonomy databases were used to create a reference database for species‐level classification of clustered OTUs. Rarefaction curves and calculations of richness and diversity indices were performed using QIIME2 (v.2019.4) [81] based on OTU data. Heatmaps displaying the top 15 OTUs were generated using Mothur to compare community membership and structure across different samples. Taxonomic annotation results, relative abundance, and OTU data were further analyzed using R software for bar plot analysis, clustering analysis, and PCA. The Bray‐Curtis method was used to calculate evolutionary distances between microbial communities in each sample, which were visualized as a clustering tree using the weighted pair group method with arithmetic mean.

### 4.31 Statistical Analysis

The reported values are presented as mean ± s.d. Comparisons among more than two groups were made using one‐way ANOVA or Kruska‐Wallis test. All of the statistical details for each experiment are described in the Figure legends. The significance was indicated in the figures, with non‐significant results (*p* > 0.05) labeled as n.s. Statistical analyses were performed using GraphPad Prism 8.0 software.

## Author Contributions

H.B. and J.J. performed conceptualization. Y.B. and X.H. performed methodology. Y.B., H.H., X.H., S.H., and Q.T. performed investigation. J.X. performed visualization. H.B. and J.J. performed supervision. Y.B, H.B., J.J., and X.H. wrote the final manuscript.

## Ethics Statement

Animal studies were approved by the Institutional Animal Care and Use Committee (IACUC) of Nanjing Medical University (IACUC Approval No. 2005036) and were conducted in accordance with international standards for animal welfare and institutional guidelines.

## Conflicts of Interest

The authors declare no conflicts of interest.

## Supporting information




**Supporting File**: advs76080‐sup‐0001‐SuppMat.docx.

## Data Availability

The data that support the findings of this study are openly available in NCBI Sequence Read Archive (SRA) at https://www.ncbi.nlm.nih.gov/sra, reference number PRJNA1159750.
