# Supplementary material for: Multi‐Targeting Carnosic Acid Kills Drug‐Resistant Helicobacter pylori With Narrow‐Spectrum Activity
Source: Adv Sci (Weinh). 2026 Jun 11;13(50):e76080. doi: 10.1002/advs.76080 (PMC13336857; doi:10.1002/advs.76080)
Supplement: Supplementary file 1 [file ADVS-13-e76080-s001.docx]

Supplementary Information for

**Multi-Targeting Carnosic Acid Kills Drug-Resistant *Helicobacter pylori* with Narrow-Spectrum Activity**

Yuefan Bai,^1,2,3,#^ Hongming Huang,^1,#^ Xudong Hang,^1,#^ Shanwei Hu^2^, Qian Tong^2^, Jingchen Xu,^2^ Jia Jia,^4,*^ Hongkai Bi^1,2,*^

^1^NHC Key Laboratory of Tropical Disease Control, School of Life Sciences and Medical Technology, Hainan Medical University, Haikou, Hainan 571199, China

^2^Helicobacter pylori Research Center, Department of Pathogen Biology, Jiangsu Key Laboratory of Pathogen Biology, Nanjing Medical University, Nanjing 211166, China

^3^Hospital for Skin Diseases, Institute of Dermatology, Chinese Academy of Medical Sciences & Peking Union Medical College, Nanjing, Jiangsu 210042, China.

^4^Deparment of Immunology and Pathogen Biology, Research Centre for Infection and Immunity, School of Medicine, Tongji University, Shanghai 200331, China.

^#^ These authors contribute equally to this work.

*Correspondence: Dr. Hongkai Bi, E-mail: hkbi@muhn.edu.cn; Dr. Jia Jia, E-mail: jiajia25@tongji.edu.cn

**Table S1.** Broth microdilution MICs (μg mL^-1^) of CA against *H. pylori* strains.

| Strains | MIC [μg mL^-1^] | | | | |
| --- | --- | --- | --- | --- | --- |
|  | CA^i)^ | LEV^ii)^ | MTZ^iii)^ | CLR^iv)^ | AMX^v)^ |
| G27 | 16 | 0.25 | 2 | 0.002 | 0.031 |
| NSH57 | 16 | 0.5 | 2 | 0.002 | 0.031 |
| 26695 | 8 | 0.25 | 1 | 0.002 | 0.031 |
| Hp159 | 8 | 8 | 16 | 0.5 | 0.031 |
| Hp129 | 8 | 8 | 16 | 0.5 | 0.031 |
| BYES00465D | 8 | 8 | 16 | 0.5 | 0.063 |
| YRES1226D | 8 | 8 | 16 | 2 | 0.25 |
| YRES874 | 4 | 4 | 32 | 0.016 | 0.125 |
| YRES761 | 8 | 0.25 | 32 | 0.016 | 0.031 |
| YR882 | 16 | 4 | 32 | 0.125 | 0.031 |
| YR01002D | 16 | 8 | 4 | 0.125 | 0.5 |
| JRES00017 | 8 | 8 | 4 | 0.063 | 0.125 |
| JRES00015 | 16 | 16 | 32 | 2 | 0.5 |
| JIGC00400 | 16 | 2 | 4 | 0.031 | 0.031 |
| JIGC00027 | 8 | 8 | 32 | 2 | 0.5 |
| JIGC00374 | 16 | 0.25 | 16 | 2 | 0.063 |
| JIGC00352 | 8 | 4 | 4 | 0.5 | 0.031 |
| JI545 | 16 | 1 | 32 | 0.063 | 0.063 |
| JIGC00341 | 8 | 0.25 | 32 | 0.031 | 0.063 |
| BYES00424 | 16 | 0.125 | 16 | 0.016 | 0.031 |
| YRES1295D | 8 | 8 | 32 | 1 | 0.031 |
| YRES829 | 8 | 0.5 | 8 | 0.016 | 0.031 |
| JI5002 | 8 | 1 | 2 | 0.063 | 0.031 |
| BY939 | 16 | 8 | 16 | 0.016 | 0.063 |
| YRES889 | 8 | 0.5 | 16 | 0.125 | 0.125 |
| YR1104 | 8 | 0.25 | 2 | 0.016 | 0.031 |
| JIGC00333 | 16 | 16 | 8 | 2 | 0.063 |
| BY583 | 16 | 4 | 8 | 1 | 0.031 |
| JRES0009 | 16 | 16 | 16 | 0.25 | 0.063 |
| BY924 | 4 | 8 | 16 | 2 | 0.063 |
| JIGC00393 | 16 | 4 | 32 | 0.5 | 0.031 |
| JIGC549 | 16 | 4 | 2 | 0.063 | 0.25 |
| JIGC00365 | 8 | 0.5 | 2 | 0.031 | 0.031 |
| YR1228 | 16 | 0.5 | 32 | 2 | 0.031 |
| YRES731 | 8 | 0.5 | 8 | 1 | 0.031 |
| BYES00388 | 16 | 1 | 16 | 2 | 1 |
| HN68 | 16 | 16 | 8 | 2 | 0.031 |
| HN32 | 8 | 8 | 16 | 2 | 0.031 |
| BNY259 | 16 | 0.125 | 1 | 0.016 | 0.031 |
| HN114 | 16 | 4 | 16 | 0.016 | 0.031 |
| JIGC00235 | 8 | 0.125 | 8 | 2 | 0.063 |

^i)^CA, Carnosic acid; ^ii)^LEV, Levofloxacin; ^iii)^MTZ, Metronidazole; ^iv)^CLR, Clarithromycin; ^v)^AMX, Amoxicillim.

**Figure S1.** Chemical structures of eight rosemary extracts.

**Figure S2.** The half maximal inhibitory concentration (IC_50_) curve of four rosemary extracts against *H. pylori* urease.

**Figure S3.** Effect of CA on the morphology and function of *E. coli* and *P. aeruginosa* cell membranes. (A) PI uptake assay of *E. coli* ATCC 25922 and *P. aeruginosa* PAO1 after treatment with CA. (B) TEM observation of *E. coli* ATCC 25922 and *P. aeruginosa* PAO1 following CA treatment. PMB was used as a positive control for membrane disruption.

**Figure S4.** Fluorescence (left) and bright-field (right) microscopy images of *H. pylori* G27 stained with CM-H_2_DCFDA after incubation with DMSO, amoxicillin (AMX), or CA. Scale bar, 10 μm.

**Figure S5.** Systemic safety evaluation of CA in mice. (A-B) Body weight of female (A) and male (B) mice following CA treatment. (C) Evaluation of the effect on red blood cells by hemolysis assay. (D-I) Hematological parameters including white blood cell count (WBC) (D), neutrophil count (Neu) (E), lymphocyte count (Lym) (F), red blood cell count (RBC) (G), hemoglobin (HGB) (H), and platelet count (PLT) (I). (J–M) Serum biochemical parameters including alanine aminotransferase (ALT) (J), aspartate aminotransferase (AST) (K), blood urea nitrogen (BUN) (L), and creatinine (Cr) (M).

**Figure S6.** (A) Weighted UniFrac distance analyses of the three groups. Each dot represents an individual sample. (B) Relative abundances of bacterial families identified in each sample from the sequencing data. Data were clustered by sample group along the x-axis.
